# Pharmacological interventions for the management of cystinuria: a systematic review

**DOI:** 10.1007/s40620-023-01795-6

**Published:** 2023-11-13

**Authors:** Nirmal Prasad Bhatt, Aniruddh Vijay Deshpande, Malcolm Ronald Starkey

**Affiliations:** 1 Department of Immunology, Central Clinical School, Monash University, Melbourne, VIC Australia; 2 Bladder and Kidney Health Discovery Program, Central Clinical School, Monash University, Melbourne, VIC Australia; 3 Centre for Kidney Research, Children’s Hospital at Westmead, New South Wales, Australia; 4 Department of Surgery, Urology Unit, Children’s Hospital at Westmead, Sydney, NSW Australia

**Keywords:** Cystinuria, Cystine, Cystine stone, Intervention

## Abstract

**Background:**

Cystinuria is a rare genetic kidney stone disease, with no cure. Current treatments involve lowering urinary cystine levels and increasing cystine solubility. This systematic review evaluates the available literature regarding non-surgical interventions for cystinuria.

**Methods:**

Key electronic databases were searched for studies that described the clinical management of cystinuria with high diuresis, alkalinizing agents and thiol-based drugs that were published between 2000 and 2022. Observational studies were included if they contained clinical investigation with at least one previous or current episode of cystine stones, urine cystine levels > 250 mg/L and patients being managed with urinary dilution, alkalinizing agents or other pharmacological agents. All included studies were assessed for study design, patient characteristics and outcomes. A qualitative and critical analysis was performed whereby study quality was assessed using Methodological Index for Non-Randomized Studies (MINORS). Two authors performed the quality assessment and excluded the studies with a low MINORS score.

**Results:**

Fourteen studies met the review inclusion and quality criteria. Of the fourteen studies, two reported treatment using alkalinizing agents, six reported treatment using thiol-based drugs, and six reported combination treatment using alkalinizing agents and thiol-based drugs. These studies indicated that first-line therapies, including high fluid intake and urinary alkalinization, increased urine volume to > 3 L/day and urinary pH > 7.0, and were associated with reduced urinary cystine levels and cystine stone formation. Second-line therapy with cystine-binding thiol drugs, such as tiopronin and D-penicillamine, reduced urinary cystine levels, cystine crystal volume and increased cystine solubility, resulting in decreased cystine stone formation and stone recurrence rate. Further, combined intervention with alkalinizing agents and thiol-based drugs synergistically reduced stone recurrence.

**Conclusion:**

Cystinuria treatment may require a combined approach of high diuresis, alkalinization and pharmacological interventions with regular monitoring of urinary pH, cystine levels, cystine crystal volume and solubility. However, poor adherence to treatment is relatively frequent, hence the pressing urgency for improved therapies and treatments.

**Graphical abstract:**

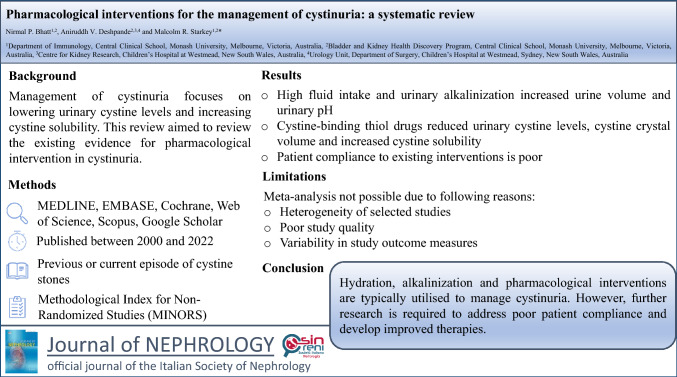

## Introduction

Cystinuria is a rare autosomal recessive condition that accounts for up to 2% of all kidney stone cases in adults and up to 8% in pediatric patients [1–6]. The estimated global prevalence of cystinuria is 1 in 7000 births, with substantial demographic differences ranging from a prevalence of 1 in 2500 births in Jews of Libyan origin and 1 in 100,000 births in Sweden [2, 7]. Cystinuria is characterized by defects in the cystine transport, primarily in the proximal renal tubules, which results in the accumulation of cystine in the urine and the formation of cystine stones [8]. The early age of disease onset, the high rate of cystine stone recurrence, and poor patient compliance with existing interventions make cystinuria a challenging condition to manage [9–11].

Cystinuria occurs due to mutations in the solute carrier family 3 member 1 (*SLC3A1*) or the solute carrier family 7 member 9 (*SLC7A9*) gene that encodes the components of the cystine transporter. *SLC3A1* gene encodes the neutral and basic amino acid transport protein (rBAT) heavy subunit [12, 13]. *SLC7A9* gene encodes the light subunit b^0,+^ type amino acid transporter 1 (b^0,+^AT) [13, 14]. Pathogenic mutations may occur simultaneously in *SLC3A1* and *SLC7A9* genes, but it is unlikely [13, 15].

Despite advances in understanding the genetic causes of cystinuria [13, 16, 17], there is currently no cure. First-line therapies for the management of cystinuria involve urinary dilution, alkalinization with potassium citrate and sodium bicarbonate, as well as limiting dietary animal protein intake, including methionine and cysteine, contributing to higher urinary pH and decreasing cystine substrate load, which helps cystine to dissolve more readily [18–20]. Pharmacological cystine-binding thiol drugs, such as alpha-mercapto propionyl glycine (tiopronin) and D-penicillamine are prescribed as second-line therapies when the first-line of therapies fail to manage cystinuria [21]. The field is currently limited by the lack of randomized controlled trials (RCTs) in cystinuria patients with follow-up periods greater than one year. Most existing studies report combined intervention approaches such as urinary dilution, alkalinization and cystine-binding thiol drug treatment before and after surgical removal of cystine stones.

This systematic review evaluates the existing literature regarding diuresis, alkalinization and pharmacological intervention strategies in the management of cystinuria and their effect on urinary pH, urinary cystine levels, urinary cystine crystal volume, urinary cystine solubility, stone-free rates and stone recurrence rates. In addition, biochemical parameters such as urinary sodium, potassium and citrate, and serum creatinine levels were also evaluated to correlate the risk of cystine stone formation.

## Methods

### Literature search

A literature search was conducted on key electronic databases, including MEDLINE, EMBASE, Cochrane, Web of Science, Scopus and Google Scholar. English language articles published between 2000 and December 2022 were included. In addition, a manual search of key journals and conference proceedings was performed to retrieve additional relevant articles. The following search terms were used individually or in combination; cystinuria, cystine stone, cystine calculi, cystine urolithiasis, cystine nephrolithiasis, cystine, kidney, cystine urine and cystinuria patient. The search terms were defined based on the PICO definition; P (population), I (intervention), and O (outcomes), while C (comparison) was only applied where placebo-control and internal control were available in the literature.

### Inclusion and exclusion criteria

Observational studies were included if they contained clinical investigation with at least one previous or current episode of cystine stones, urine cystine levels > 250 mg/L and patients being managed with urinary dilution, alkalinizing or pharmacological agents. There were no exclusions based on age, gender, ethnicity, or geographical location and follow-up of included studies. Abstracts, pilot studies and clinical trials were included if there was sufficient data, and corresponding authors were contacted to obtain full study details. Review articles, editorials, news, letters, comments, case series and case reports were excluded. However, review articles were used for cross-referencing to retrieve any missing studies and did not contribute to the final number of articles. The inclusion and exclusion criteria were independently applied to all identified articles. Two authors (NPB and MRS) participated in the initial screening of titles and abstracts and independently screened the titles and abstracts before assessing full-text articles. Multiple author agreements resolved any disputes. We contacted the authors of the primary reports to request any unclear or unpublished data. If the authors did not reply, the available data was used.

### Data extraction

This systematic review follows Preferred Reporting Items for Systematic Reviews and Meta-Analysis (PRISMA) guidelines [22]. References from the database search were pooled into Endnote 20 reference software. Selected data were assessed for study design, aims, inclusion criteria, patient characteristics, intervention strategy and primary outcomes, including changes in urinary pH, cystine levels, cystine crystal volume, cystine solubility, and cystine stone-free and stone recurrence rates after the interventions. Secondary outcomes such as urinary sodium, potassium, citrate, and serum creatinine levels were observed to correlate with the effectiveness of the intervention. Dilution, alkalinization and pharmacological outcomes of intervention were compared with baseline patient history or the control group. The study protocol was registered with PROSPERO to guide this systematic review (ID: CRD42020152061).

### Risk of bias (quality) assessment

Most of the available quality assessment tools are designed to evaluate RCTs, case–control or cohort studies. The methodological quality assessment tool, Methodological Index for Non-Randomized Studies (MINORS), was used to assess the studies that met the inclusion criteria. MINORS is a validated tool for the methodological quality assessment of non-comparative and comparative observational studies [23]. It consists of eight items for non-comparative and an additional four items for comparative studies. Two authors performed the quality assessment independently (NPB and MRS) and excluded the studies with a low MINORS score. In addition, AVD cross-checked the selected literature and quality assessment tools to resolve disputes.

### Data synthesis

Due to the anticipated variability in the included studies, and the lack of RCTs for this rare disease, a meta-analysis was not planned. Hence, a qualitative and critical data analysis was performed with available patient characteristics, disease history, and treatment procedures and effectiveness. In addition, post-treatment outcomes, as mentioned above, were compared with the patient’s history.

## Results

### Search results

**Fig. 1:**
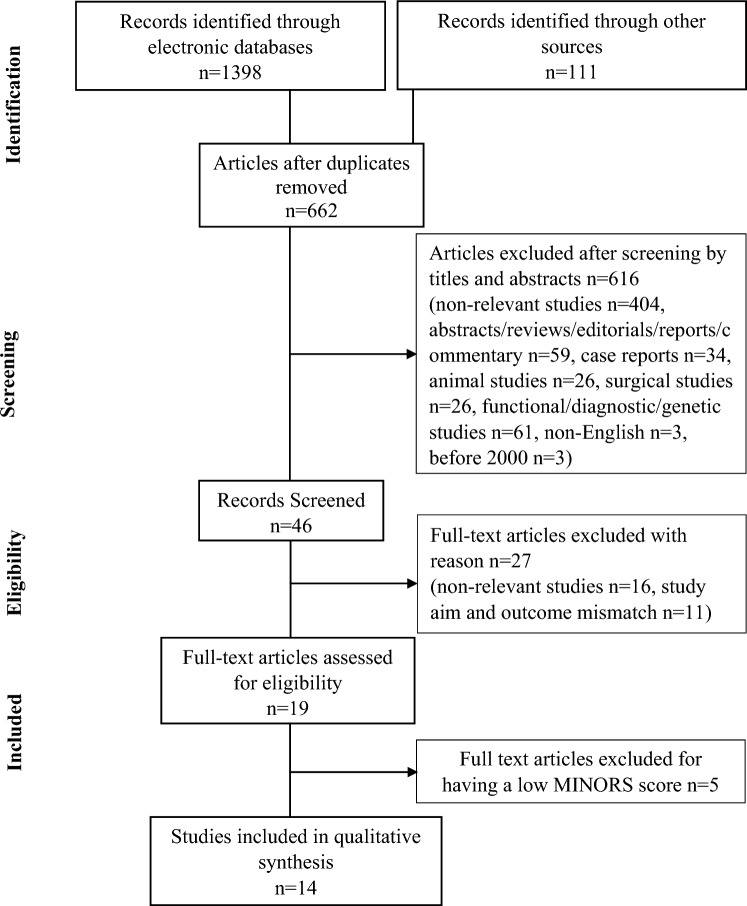
PRISMA flow diagram of the literature search

The initial database (MEDLINE, EMBASE, Cochrane, Web of Science, Scopus and Google Scholar) and Google search retrieved a total of 1509 articles. After excluding duplicate studies, 662 were reviewed for title and abstract screening. We excluded 616 due to being non-relevant (404), abstracts/reviews/editorials/reports/commentary (59), case reports (34), animal studies (26), surgical studies (26), functional/diagnostic/genetic characterization studies (61), non-English (3) or published before 2000 (3). A total of forty-six studies underwent full-text review. After screening the complete text, 27 articles were excluded for the following reasons, non-relevant studies (16), or inappropriate study aims and outcomes (11). Nineteen studies met the review criteria, and the MINORS quality assessment tool was applied to these studies, which resulted in the exclusion of five studies that did not meet the quality requirements (Fig. 1).

### Study characteristics

Three hundred and forty-nine cystinuria patients (an average of 24.9 per study) were included in this review. Among these patients, 161(52%) were males, and 150 (48%) were females from eleven studies [11, 19, 20, 24–31]. Three studies did not specify the patient’s gender [32–34]. Thirteen studies reported the mean patient age ranging from 2.5 to 49 years [19, 20, 24–32, 34, 35]. Nine studies were prospective observational, and five studies were retrospective observational. Treatment outcomes were compared with either the control group or patient baseline characteristics. Of the fourteen studies, three were non-comparative, while eleven were comparative. A summary of the study characteristics is provided in Table 1. The quality of the included studies was assessed using the MINORS score. The mean score for non-comparative studies was 11 out of 16 (range, 8–13). For non-comparative studies, MINORS scores were classified as; low (score 5–8), moderate (score 9–12), high quality (score 13–16) (Table 2). The mean score for comparative studies was 16 out of 24 (range, 12–21). For comparative studies, MINORS scores were classified as; low (score 7–12), moderate (score 13–18), high quality (score 19–24) (Table 3). Those studies that scored a moderate or high MINORS score were included. Five studies were excluded due to a low MINORS score.

**Table 1:** Characteristics of included studies

Study reference (alphabetical) and study type	Number of patients (male (M) and female (F), or unknown (U) and mean or median age (years)	Fraction of patients (alakalinization and pharmacological treatment)	Mean follow-up period	Compliance to treatment (Yes/Partial)	Stone recurrence rate (%)	Stone-free rate (%)	Alkalinization and/or thiol drug success (Yes/Partial)
Asi et al. 2020 [24] Retrospective observational	70 (40 M and 30F) pediatric cystinuria patients Mean age = 2.5 yrs	70/70 alkalinization, 52/70 Tiopronin or 18/72 Captopril	1 year	Partial	71	47	Yes
Barbey et al*.* 2000 [19] Retrospective observational	27 (12 M and 15F) cystinuria patients Mean age = 19.6 yrs	5/27 Potassium citrate or 22/27 Sodium bicarbonate, (8–18 g/day), 3/27 Tiopronin (500–1000 mg/day) or 6/27 D-penicillamine (600-1200 mg/day) or 3/27 Captopril (100–150 mg/day)	11.6 years	NR	45	48	Yes
Daudon et al*.* 2003 [25] Prospective observational	57 (29 M and 28F) cystinuria patients Mean age = 25.8 yrs	6 Potassium citrate (60-80 mEq/day) or 46/57 Sodium bicarbonate (8–18 g/day according to body weight), Tiopronin (500–1500 mg/day or D-penicillamine (600–1200 mg/day) or 6/57 Captopril (100–150 mg/day)	1 year	NR	47	53	Yes
DeBerardinis et al. 2008 [32] Retrospective observational	11 (U) cystinuria patients Mean age = 7.4 yrs	11/11 D-penicillamine (5-20 mg/kg/day)	9 years	Yes	NR	NR	Partial
Dolin et al. 2005 [34] Prospective observational	7 (U) cystinuria patients Mean age = 41.6 yrs	5/7 Tiopronin (between 600 and 2500 mg/day) or 2/7 D-penicillamine (between 1000 and 2500 mg/day) or 1/7 Captopril (150 mg/day)	10 days	Yes	NR	NR	Yes
Fjellstedt et al. 2001 [20] Prospective observational	14 (8 M and 6F) cystinuria patients Mean age = 21 yrs	13/14 Potassium citrate (60 mmol/day) or 1/14 Sodium bicarbonate (71.4 mmol/day) or 10 Tiopronin (1250–3000 mg/day)	0.1 years	Yes	NR	NR	Yes
Izol et al. 2013 [26] Prospective case–control	17 (10 M and 7F) cystinuria patients Mean age = 8.5 yrs	17/17 Shohl’s solution (Potassium citrate and citric acid) (2 mmol/kg/day in 3 doses)	2.5 years	Partial	41	NR	Yes
Malieckal et al. 2019 [27] Prospective observational (randomized controlled trial)	10 (7 M and 3F) cystinuria patients Mean age = 49 yrs	7/10 Tiopronin (0–3 g/day) or 3/10 D-penicillamine (0–3 g/day)	4 weeks	Partial	NR	NR	Yes
Mohammadi et al*.* 2018 [28] Prospective observational (clinical trial)	48 (23 M and 25F) cystinuria patients Mean age = 39.2 yrs	48/48 Selenium (200 mg/day)	6 weeks	Yes	NR	NR	Yes
Nelson et al*.* 2020 [29] Prospective observational (pilot study)	4 (2 M and 2F) cystinuria patients Mean age = 19.3 yrs	4/4 Tolvaptan (0.3 mg/kg/day, maximum 30 mg to 0.6 mg/kg/day, maximum 60 mg)	8 days	Yes	NR	NR	Yes
Pareek et al. 2005 [30] Retrospective observational	20 (6 M and 14F) cystinuria patients Mean age = 45 yrs	Tiopronin or D-penicillamine or Captopril	3.5 years	Partial	NR	55	Yes
Pietrow et al. 2003 [31] Retrospective observational	26 (11 M and 15F) cystinuria patients Mean age = 32 yrs	26/26 Tiopronin (1000 mg/day)	3.2 years	Partial	NR	NR	Partial
Strologo et al. 2007 [33] Prospective observational	20 (U) cystinuria patients Mean age = 12.6 yrs	10/20 Potassium citrate or 9/20 Sodium bicarbonate, 16/20 Tiopronin (25 mg/kg/day) or 2/20 D-penicillamine (17 mg/kg/day)	3.5 years	Partial	NR	NR	Yes
Tekin et al*.* 2001 [11] Prospective case–control	18 (13 M and 5F) cystinuria patients versus 24 (17 M and 7F) healthy cohorts Median age = 6.5 yrs	18/18 Potassium citrate (1 mEq/kg/day), 18/18 Tiopronin (10-15 mg/kg/day)	1.3 years (median)	Yes	28	56	Yes

*NR* not reported, *U* unknown

**Table 2:** Quality assessment for included non-comparative studies

Items	Study		
	Barbey et al. 2000 [19]	DeBerardinis et al. 2008 [32]	Strologo et al. 2007 [33]
A clearly stated aim (/2)	2	2	2
Inclusion of consecutive patients (/2)	2	2	2
Prospective collection of data (/2)	2	0	1
Endpoints appropriate to the aim of the study (/2)	2	2	2
Unbiased assessment of the study endpoint (/2)	1	1	1
Follow-up period appropriate to the aim of the study (/2)	2	2	2
Loss to follow-up less than 5% (/2)	1	1	0
Prospective calculation of the study size (/2)	0	0	0
TOTAL SCORE (/16)	12	10	10
Study quality	M	M	M

*M* moderate score (*M* = 9–12)

**Table 3:** Quality assessment for included comparative studies

Items	Study										
	Asi et al. 2020 [24]	Daudon et al. 2002 [25]	Dolin et al. 2005 [34]	Fjellstedt et al. 2001 [20]	Izol et al. 2013 [26]	Malieckal et al. 2019 [27]	Mohammadi et al. 2018 [28]	Nelson et al. 2020 [29]	Pareek et al. 2005 [30]	Pietrow et al. 2003 [31]	Tekin et al. 2001 [11]
A clearly stated aim (/2)	2	2	2	2	2	2	2	2	2	2	2
Inclusion of consecutive patients (/2)	2	2	1	2	2	2	2	1	2	2	2
Prospective collection of data (/2)	0	2	2	1	1	2	2	2	2	0	1
Endpoints appropriate to the aim of the study (/2)	1	2	2	1	2	2	2	2	2	2	2
Unbiased assessment of the study endpoint (/2)	2	1	2	2	1	2	2	1	2	1	2
Follow-up period appropriate to the aim of the study (/2)	2	2	0	1	2	0	1	0	2	2	1
Loss to follow-up less than 5% (/2)	1	1	2	1	2	0	2	2	2	1	2
Prospective calculation of the study size (/2)	0	0	0	0	0	0	1	0	0	0	0
An adequate control group (/2)	1	1	1	2	1	0	1	1	1	0	2
Contemporary groups (/2)	1	0	0	1	2	2	1	0	2	1	2
Baseline equivalence of groups (/2)	1	0	1	1	1	2	2	1	2	2	1
Adequate statistical analysis (/2)	1	2	1	2	1	1	1	1	2	2	1
Total score (/24)	14	15	14	16	17	15	19	13	21	15	18
Study quality	M	M	M	M	M	M	H	M	H	M	M

*M* moderate score (*M* = 13–18)

Of the fourteen studies, two reported outcomes of first-line therapy using urinary dilution and alkalinizing agents (potassium citrate and sodium bicarbonate) [20, 26]. Six studies reported second-line therapy outcomes from treatment with pharmacological compounds (tiopronin, D-penicillamine, captopril or tolvaptan) [27, 29–32, 34]. Further, six of the fourteen studies reported a combined treatment approach of urinary dilution, alkalinization and thiol-based drugs [11, 19, 20, 24, 25, 33].

### First-line therapy

Initial management of cystinuria includes maintaining daily urine volume > 3 L/day [17]. High fluid intake along with alkalinizing compounds was used to sustain urinary pH > 7.0, contributing to the increase in the solubility of cystine in the urine [20]. Seven of the fourteen studies reported using potassium citrate or sodium bicarbonate as alkalinizing agents for cystinuria management [11, 19, 20, 24–26, 33]. Only five studies reported the doses of alkalinizing agents and their treatment outcomes (Table 4) [11, 19, 20, 25, 26]. Potassium citrate (60–80 mEq/day [25] or 1 mEq/kg/day [11] or 40 to 70 mmol/day[20]) and sodium bicarbonate (8–18 g/day [19, 25] according to body weight or 47.6–107 mmol/day [20]) were used to increase urinary pH levels by increasing the excretion of free bicarbonate ions without producing systemic alkalosis [36].

**Table 4:** Effectiveness of diuresis and alkalinization

Study reference	Interventions	pH change	Mean urinary cystine levels or urinary cystine volume	Urinary sodium/potassium/citrate or serum creatinine levels	Key outcomes
Barbey et al. 2000 [19]	Potassium citrate or Sodium bicarbonate (8–18 g/day according to body weight)	Increased to 7.5	Cystine levels at baseline 798 ± 304 mg/24 h to 808 ± 305 mg/24 h after treatment	Urinary sodium: 173 ± 58 mmol at baseline to 263 ± 91 mmol daily, *p* < 0.001 after treatment Urinary citrate: 2.9 ± 1.1 mmol at baseline to 5.4 ± 1.3 mmol daily after treatment, *p* < 0.01 Serum creatinine: 90 ± 15 at baseline to 94 ± 19 µmol/L after treatment	Increased urinary pH, sodium, and citrate levels in treatment group
Daudon et al. 2003 [25]	Potassium citrate (60-80 meq/day) or Sodium bicarbonate (8-18 g/day according to body weight)	NR	Cystine volume decreased from 12,097 ± 3214 μ^3^/mm^3^ at baseline to 2,648 ± 658 μ^3^/mm^3^ on hydration and alkalinization therapy, *p* < 0.05	NR	Reduced urinary cystine volume in treatment group
Fjellstedt et al. 2001 [20]	Potassium citrate (40-70 mmol/day or Sodium bicarbonate (47.7-107 mmol/day)	6.4 to 7.10, *p* < 0.05 on potassium citrate and 6.4 to7.25, *p* < 0.01 on sodium bicarbonate	NR	Urinary sodium: 144 to 220 mmol 24 h, *p* < 0.05 Urinary potassium: 63 mmol at baseline to 94 mmol 24 h after treatment, *p* < 0.01	Increased urinary pH levels treatment group
Izol et al. 2013 [26]	Shohl’s solution (Potassium citrate and Citric acid) (2 mmol/kg/day in 3 doses)	5.8 in pretreated and 7.5 in post-treated patients, *p* < 0.001	NR	NR	Increased urinary pH levels in treatment group
Tekin et al. 2001 [11]	Potassium citrate (1 mEq/kg/day)	Up to 7.5	NR	Urinary citrate: 255 ± 219 mg./1.73/m^3^ at baseline to 729 ± 494 mg./1.73/m^3^ after treatment, *p* = 0.003	Increased urinary pH and urinary citrate levels in treatment group

*NR* not reported

Potassium citrate and sodium bicarbonate were effective in alkalinizing the urine. Urinary pH levels increased from 6.4 to 7.10, *p* < 0.05 and 5.6 ± 0.2 to 6.9 ± 0.3, *p* = 0.02, and from 6.4 to 7.25, *p* < 0.01, respectively, compared to baseline measurements [11, 20]. Potassium citrate or sodium bicarbonate doses of 8 to 18 g/day were effective in increasing the urinary pH up to 7.5, which inhibits cystine stone formation [19]. An alkalinizing compound, Shohl’s solution was found to increase pH levels (5.8 ± 0.5 to 7.5 ± 0.4, *p* < 0.001) compared to pre-treatment measurements [26]. Urinary cystine crystal volume (12,097 ± 3214 to 2648 ± 658 μ^3^/mm^3^, *p* < 0.05) was significantly decreased with potassium citrate treatment (60 to 80 meq/day) or sodium bicarbonate (8–18 g/day according to body weight) compared to baseline measurements [25].

Other biochemical parameters reported were also related to increasing urinary pH and reducing cystine concentration. Urinary citrate levels were significantly higher in cystinuria patients (491 ± 490 and 262 ± 428 mg/1.73/m^3^, *p* = 0.044) compared to healthy cohorts [11]. Urinary citrate levels were also significantly increased by potassium citrate or sodium bicarbonate treatment (2.9 ± 1.01–5.4 ± 1.3 mmol/day, *p* < 0.01) compared to baseline measurements [19]. In addition, urinary citrate (*p* < 0.05) and plasma citrate levels (3.7 to 4.3 mmol/L, *p* < 0.01) both significantly increased during treatment with potassium citrate compared to baseline measurements [20]. Urinary potassium levels were also significantly increased (63–94 mmol/day, *p* < 0.01) during treatment with potassium citrate compared to baseline measurements [20]. Likewise, urinary sodium levels were significantly increased (144–220 mmol/day, *p* < 0.05 and 173 ± 58–263 ± 91 mmol/day, *p* < 0.001) with sodium bicarbonate treatment compared to baseline measurements [19, 20]. Urinary cystine levels may be controlled by limiting cysteine and methionine in the diet. However, this was inadequately reported for inclusion in the systematic analysis.

### Second-line therapy

Ten studies reported cystinuria interventions using thiol-based drugs; tiopronin, D-penicillamine and captopril [11, 19, 24, 25, 27, 30–34]. One study used selenium, and one used the vasopressin antagonist tolvaptan [28, 29]. These pharmacological interventions were typically recommended when diuresis and alkalinization treatment was insufficient to increase urinary pH and decrease urinary cystine levels [21]. Various concentrations of tiopronin (500–2500 mg/day [19, 25, 31, 34] or 10–25 mg/kg/day [11, 33]), D-penicillamine (600–2500 mg/day [19, 25, 34] or 5–20 mg/kg/day [32, 33]), and rarely captopril (100–150 mg/day [25]) were used to treat cystinuria patients. Urinary pH, cystine levels, cystine crystal volume, cystine solubility, and stone-free and stone recurrence rates were reported during follow-up of cystinuria patients (Table 5).

**Table 5:** Effectiveness of pharmacological interventions

Study reference	Interventions	pH change	Mean follow-up time	Mean urinary cystine levels or urinary cystine volume	Urinary cystine solubility	Key outcomes
Asi et al. 2020 [24]	Tiopronin and Captopril	NR	1 year	Cystine levels: 233 ± 106.9 mg/L in compliant and 479.6 ± 534.5 mg/L in non-compliant patients, *p* = 0.028	NR	Decreased urinary cystine levels in treatment compliance group
Barbey et al. 2000 [19]	D-penicillamine (600-1200 mg/day), Tiopronin (500-1000 mg/day), Captopril (100-150 mg/day)	7.6 ± 0.6 in therapeutic success and 7.5 ± 0.5 in therapeutic failure patients, NS	11.6 years	Cystine levels: 857 ± 149 mg/24 h at baseline to 585 ± 128 mg/24 h after treatment,* p* = 0.046	NR	Decreased urinary cystine levels in treatment group
Daudon et al. 2003 [25]	Tiopronin (500–1500 mg/day, D-penicillamine (600–1200 mg/day), Captopril (100-150 mg/day)	NR	1 year	Cystine volume: 12,097 ± 3214 μ^3^/mm^3^ at baseline to 1141 ± 522 μ^3^/mm^3^ on tiopronin and 791 ± 390 μ^3^/mm^3^ on D-penicillamine treatment*, p* = 0.01	NR	Decreased urinary cystine crystal volume in treatment group
DeBerardinis et al. 2008 [32]	D-penicillamine (5-20 mg/kg/day)	NR	9 years	Cystine levels: decreased by 54% after treatment	NR	Decreased urinary cystine levels in treatment group
Dolin DJ et al. 2005 [34]	Tiopronin (between 600 and 2500 mg/day), D-penicillamine (between 1000 and 2500 mg/day), Captopril (150 mg/day)	6.89 ± 0.43 on thiol drugs and 7.04 ± 0.27 off thiol drugs, NS	10 days	NR	Increased cystine capacity from − 130.6 mg/L to 43.1 mg/L, *p* < 0.05	Increased cystine solubility in treatment group
Malieckal et al. 2019 [27]	Tiopronin (0-3 g/day), D-penicillamine (0-3 g/day)	NR	NR	Cystine levels: 1003.9 mg/24 h at baseline to 834.8 mg/24 h after treatment, *p* < 0.039	Increased cystine capacity from − 39.1 mg/L at baseline to 130.4 mg/L, *p* < 0.009	Decreased urinary cystine levels and increased cystine solubility in treatment group
Mohammadi et al. 2018 [28]	Selenium (200 mg/day)	NR	6 weeks	Cystine volume: 6787.4 ± 11,902.6 μ^3^/mm^3^ pre-treatment and 3110.9 ± 7225.4 μ^3^/mm^3^ after treatment, *p* < 0.001	NR	Decreased urinary cystine crystal volume in treatment group
Nelson et al. 2020 [29]	Tolvaptan (0.3 mg/kg/day-0.6 mg/kg/day)	NR	8 days	NR	Increased cystine capacity from − 344 mg/L at baseline to 70 mg/L on treatment	Increased cystine solubility and urinary output volume
Pareek et al. 2005 [30]	Tiopronin or D-penicillamine, Captopril	7.4 ± 0.2 in compliant and 7.1 ± 0.3 noncompliant patients, *p* = 0.66	3.5 years	Cystine levels: 282.2 ± 52.6 mg/L in compliant and 382.4 ± 61.3 mg/L in non-compliant patients	NR	Decreased urinary cystine levels in treatment compliant group
Pietrow et al. 2003 [31]	Tiopronin (1000 mg/day)	NR	3.2 years	Cystine levels: 154.3 mg/L in therapeutic and 422.4 mg/L in non-therapeutic group, *p* = 0.004	NR	Decreased urinary cystine levels in treatment group
Strologo et al. 2007 [33]	Tiopronin (25 mg/kg/day), D-penicillamine (17 mg/kg/day)	> 7.0 in 80% of patients	3.5 years	NR	NR	Increased pH and decreased urinary cystine levels in treatment group
Tekin et al. 2001 [11]	Tiopronin (10–15 mg/kg/day)	5.6 ± 0.2 at baseline to 6.9 ± 0.3 on treatment, *p* = 0.020	1.3 years (median)	Cystine levels: 245 ± 233 mmol/mol.creatinine in pre-treatment and 140 ± 106 mmol/mol.creatinine in post-treatment group, *p* = 0.015	NR	Decreased urinary cystine levels in treatment group

*NR* not reported

Urinary pH was significantly increased (5.6 ± 0.2–6.9 ± 0.3, *p* = 0.020) following tiopronin treatment compared to pre-treatment measurements [11]. Seven studies reported an association between pharmacological interventions, and urinary cystine levels, stone-free and stone recurrence rates [11, 19, 24, 27, 30–32]. A significant reduction in urinary cystine levels was observed between baseline and post-treatment with thiols (857 ± 149–585 ± 128 mg/24 h) [19] and between compliant and non-compliant with a thiol-treated group (282.2 ± 52.6–382.4 ± 61.3 mg/L) [30]. Urinary cystine levels were decreased by tiopronin treatment (1,052 ± 161–755 ± 81 mg/day) and by D-penicillamine treatment (789 ± 126 to 517 ± 92 mg/day); however, captopril did not change urinary cystine levels (1044 ± 57 to 1039 ± 137 mg/day) during the mean follow-up period of 11.6 years [19]. An additional two studies reported a significant reduction in urinary cystine levels (245 ± 233 to 140 ± 106 mmol/mol creatinine, *p* = 0.015) [11] and (1003.9 to 834.8 mg/day,* p* < 0.039) [27] following intervention with tiopronin or D-penicillamine compared to pre-treatment. Tiopronin alone significantly reduced urinary cystine levels in cystinuria (154.3 mg/L vs 422.4 mg/L, *p* = 0.004) compared to nontreated patients [31]. Another study revealed that the average urinary cystine levels were reduced by 54% (range, 5 to 81%) with D-penicillamine [32], and 15% of cystinuria patients maintained urinary cystine levels < 300 mg/L following tiopronin treatment [31]. When comparing pediatric and adult cystinuria patients, tiopronin and D-penicillamine significantly reduced urinary cystine levels 140 ± 106 mmol/mol creatinine after treatment in pediatric [11], and 154.3 mg/L in therapeutic adult [31] cystinuria patients. Further, combined interventions with diuresis, alkalinization and cystine-binding thiol drugs significantly decreased urinary cystine levels (808 ± 305 to 585 ± 128 mg/day, *p* = 0.046) [19] compared to baseline measurements.

Mean urinary cystine crystal volume was decreased from baseline 12,097 ± 3214–1141 ± 522 μ^3^/mm^3^ on tiopronin therapy and 791 ± 390 μ^3^/mm^3^ on D-penicillamine therapy. In contrast, captopril treatment was less effective (12,097 ± 3214–5114 ± 2,128 μ^3^/mm^3^) [25]. Selenium, an antioxidant compound, had shown a significant reduction in urinary cystine crystal volume (6787.4 ± 11,902.6 to 3110.9 ± 7225.4 μ^3^/mm^3^, *p* < 0.001) [28]. Cystine solubility was significantly increased, showing positive values from − 130.6 to 43.1 mg/L, *p *< 0.05 [34] and − 39.1 to 130.4 mg/L, *p *< 0.009 [27] with thiol interventions. A pilot study of cystinuria treatment with tolvaptan, a vasopressin compound, was also found to increase urinary cystine solubility from − 344 to 70 mg/L after treatment [29].

Urinary citrate levels were significantly increased with tiopronin treatment (255 ± 219–729 ± 494 mg/1.73/m^3^, *p* = 0.003) compared to baseline measurement [11]. Urinary sodium levels were 155.4 ± 64.8 mEq/day off cystine-binding thiol drugs and 121.5 ± 46.2 mEq/day on cystine-binding thiol drugs, and creatinine levels were 1773.9 ± 516.1 mg/day on cystine-binding thiol drugs and 1739.3 ± 377 mg/day off cystine-binding thiol drugs [34]. However, these secondary outcomes were poorly reported in the current cystinuria literature.

### Patient compliance

Two studies reported on patient compliance with follow-up protocols based on their diet, medication regimen and frequency of patient visits to the clinic following intervention [24, 30]. Both pediatric and adult cystinuria patients who were compliant with pharmacological interventions had significantly reduced urinary cystine levels (233 ± 106.9 mg/L vs 479.6 ± 534.5 mg/L, *p* = 0.028 and 282.2 ± 52.6 mg/L vs 382.4 ± 61.3 mg/L) compared to non-compliant patients [24, 30]. Additionally, pediatric cystinuria patients had a higher reduction in urinary cystine levels compared to adult cystinuria patients after pharmacological treatments [24, 30]. A monthly, quarterly and semi-annual clinic visit was required for medical evaluation of cystinuria patients after initial surgical interventions. Stone-free rates were higher for treatment-compliant patients than non-compliant patients (73% vs 33%) [30]. Decreased stone recurrence rates were reported in patients compliant with pharmacological treatment after initial interventions and a longer time to recurrence of stones compared to non-compliant patients (30.5 months vs 20.7 months, *p* = 0.047) [24].

## Discussion

In this systematic review, results were synthesized from the observational studies of existing intervention approaches for cystinuria management, which include high fluid intake, alkalinization of the urine, and pharmacological treatments. This review does not provide conclusions of the hierarchy of treatment. This has been reviewed elsewhere and is addressed in current clinical guidelines [18, 37]. All cystinuria interventions are intended to decrease urinary cystine levels and increase cystine solubility. Increased fluid intake, sodium and protein ingestion restriction, and urine alkalinization are conventional therapies for cystinuria management. Cystinuria patients require a sufficient amount of fluid intake to maintain a high urinary output to pass out cystine crystals with the urine stream. Potassium citrate and sodium bicarbonate were commonly used alkalinizing agents to treat cystinuria by increasing urinary pH and decreasing urinary cystine levels [11, 19, 20, 26]. Pharmacological interventions for cystinuria are only recommended following the failure of the first-line treatments, including fluid intake and alkalinization [21, 38]. Second-line approaches involve treatment with the thiol-based compounds, tiopronin and D-penicillamine, and rarely with the less effective captopril [11, 19, 24, 25, 27, 30–34]. Pharmacological treatments and increased fluid intake effectively lower urinary cystine levels and cystine crystal volume and increase cystine solubility, which is associated with decreased stone recurrence and increased stone-free rates [11, 19, 24, 25, 27–32, 34]. However, these interventions often fail due to poor patient compliance. Hence, combined treatment approaches, with regular clinic visits have a synergistic beneficial impact on cystine stone formation [11, 24, 30, 33].

The early treatment strategy for cystinuria is focused on reducing urinary cystine levels and increasing urinary cystine solubility. Cystine is poorly soluble at normal urinary pH, and crystals accumulate when urinary cystine concentrations exceed 250 mg/L [39]. However, managing fluid intake to maintain urinary volume > 3L/day and reducing methionine and salt intake in the diet can decrease urinary cystine levels [40]. Cystinuria patient management varies, however, lifestyle changes and using alkalinizing agents are recommended for all patients [41]. Treatment approaches using adequate fluid intake and alkalinizing agents are most effective in increasing urinary pH and reducing urinary cystine and cystine stone formation [20]. Potassium citrate and sodium bicarbonate are the most commonly used alkalinizing agents. Potassium citrate is more effective in reducing sodium concentration and increasing urine pH [20, 36, 42]. Prophylactic treatment with Shohl’s solution, a combination of potassium citrate and citric acid, also increases urinary pH levels and decreases urinary cystine concentrations [26]. Similarly, sodium bicarbonate is effective in increasing urinary pH levels [20]. Treatment with sodium bicarbonate reduces urinary cystine crystal size by inhibiting crystal agglomeration [43]. However, the use of sodium bicarbonate is controversial due to the link between the tubular reabsorption of cystine and sodium, and sodium bicarbonate may have relevant side effects including abdominal pain, nausea and vomiting. Hence, sodium bicarbonate is mainly recommended when cystinuria patients have renal insufficiency or intolerance to potassium citrate [39, 44]. In addition to high fluid intake and alkalinization of urine, cystinuria patients frequently require treatment with cystine-binding thiol drugs.

Tiopronin and D-penicillamine are the most common cystine-binding thiol drugs used for cystinuria treatment [41]. Captopril is rarely used to treat cystinuria when standard therapy using high diuresis, alkalinization and tiopronin or D-penicillamine has not been effective [40]. However, the efficacy of captopril in managing cystinuria is not extensively reported [45]. Thiol compounds contain a sulfhydryl group that undergoes a disulfide exchange with cystine to produce two cysteine molecules bound to the cystine-binding drugs. This disulfide complex is more soluble than cystine in the urine [39]. Both tiopronin and D-penicillamine had similar outcomes in decreasing urinary cystine concentration and stone formation, but the latter has been shown to have fewer side effects compared to D-penicillamine [25, 45]. Additionally, tiopronin treatment showed better health-related quality of life for cystinuria patients [46]. Pharmacological interventions have variability in efficacy, and cystinuria patients frequently experience adverse events such as gastrointestinal intolerance, while nephrotic syndrome, hepatotoxicity, rash, and leukopenia are more severe but less frequent, although all impact patient compliance [40, 47]. Hence, cystinuria patients require regular monitoring to minimize adverse events and improve health-related quality of life [35].

Further monitoring of biochemical parameters such as urinary pH, cystine levels, cystine crystal volume and cystine capacity is also required to manage cystinuria properly. Currently, Litholink, a 24-h urine test is available only in the USA [48]. All cystinuria treatments are directly or indirectly targeted to decrease urinary cystine levels and increase urinary cystine solubility. However, there is variability in reporting the outcome of pharmacological intervention and only few studies have reported biological parameters such as changes in urinary pH, cystine level, cystine crystal volume and cystine solubility, as well as stone recurrence and stone-free rates.

In addition to the urinary pH, measuring urinary cystine level is useful for cystinuria management. Urinary cystine level measurement during thiol treatment is inaccurate because thiol compounds exchange disulfide with cystine to form a complex that is more soluble in urine. Cystine quantification in 24-h urine is more useful for patients using cystine-binding thiol drugs [49]. However, separate day and night urine sampling for cystine measurements may further aid in optimizing patient-oriented cystinuria treatment [19, 20]. Another biochemical parameter, urinary cystine crystal volume, predicts the risk of cystine stone recurrence, and cystine crystal volume less than 3000µ^3^/mm^3^ is associated with a lower risk of cystine stone formation [25]. Combining alkalinization and thiol treatment increases cystine solubilization, ultimately decreasing urinary cystine levels. In the current treatment protocols, measuring the cystine crystal volume in early-morning urine to predict stone recurrence, and tiopronin and D-penicillamine treatment were found to lower the cystine crystal volume [25]. Therefore, a  solid-phase urinary cystine solubility assay is recommended to monitor the patient's response and modulate alkalinizing and thiol doses [50, 51]. It is suggested that the cystine solubility measurement is a reliable way for predicting the risk of stone formation in cystinuria patients [34]. Additional biochemical parameters such as urinary citrate, sodium, potassium and creatinine levels can be helpful for modulating pharmacologic and dietary plans. However, these parameters are rarely reported in the literature.  

 Dietary protein restriction, mainly targeted at reducing animal-derived proteins is also an important component of cystinuria management. Reducing dietary intake of cystine and methionine decreases urinary cystine levels [18, 44]. At the same time, a plant-based diet was found to be effective in increasing urinary pH levels [52]. Therefore, lowering animal protein intake is recommended to get urinary cystine levels < 250 mg/L, and it also has the potential to increase urine pH [44]. Whilst protein restriction is usually not recommended in children, methionine content in food can be minimized [44, 53].

Patient compliance to pharmacological treatment is usually poor, and non-compliance to the treatment results in higher stone recurrence and lower stone-free rates after interventions [28, 29]. Low compliance is partly due to the side effects of pharmacological agents [26]. However, timely clinic visits and medical advice could minimize stone recurrence in cystinuria patients. In addition to current pharmacologic treatments, there is a pressing need to improve management based on patient history and regular clinic visits. 

There is urgent need to develop pharmacological interventions that could reduce the current adverse effects of thiol-based drugs and improve patient compliance. Early intervention with alkalinization therapy is highly important. In addition to traditional alkalinizing agents, acetazolamide, a diuretic and carbonic anhydrase inhibitor, may replace potassium citrate, which effectively reduces cystine stone formation; however, regular clinical and laboratory examinations have been recommended to monitor adherence and tolerance [54]. A recent pilot study in four cystinuria patients reported that tolvaptan, a vasopressin antagonist, reduced the risk of stone recurrence by increasing urine output volume and cystine solubility [29]. This compound has also been used in mouse models of cystinuria, and the results showed that tolvaptan delayed the cystine stone growth by increasing urine volume [55]. Selenium, an antioxidant compound, is also effective in reducing urinary cystine crystal volume and was able to inhibit cystine crystal aggregation over a short period [28]. A long-term study of the effect of selenium supplementation in cystinuria patients is required to validate its use. 

This review has several limitations, including the heterogeneity among the available studies, retrospective data, low sample size, incomplete reporting of outcomes, short-term follow-up period. As in most rare diseases, there is a lack of RCTs on cystinuria. In this review, most studies lack proper control groups. Regular monitoring of biochemical parameters needs to be better reported to help manage cystinuria patients in terms of treatment outcomes. Indeed, RCTs would be required to validate the effectiveness of pharmacological interventions in cystinuria.

## Conclusions

This systematic review highlights that cystinuria treatment is challenging, requiring various intervention strategies, including high diuresis, alkalinization, and pharmacological treatments. The objective of treatment is to reduce urinary cystine concentration and to increase cystine solubility in the urine. Combined interventions are limited by low adherence to the extensive lifestyle changes and adverse effects of pharmacological drugs. Emerging therapies based on a better understanding of cystinuria pathogenesis and treatment should focus not only on reducing urinary cystine levels, but also on decreasing side effects and preserving renal function. Randomized controlled trials with long-term follow-up and a large sample size would ideally be the appropriate design for testing optimal treatment strategies for cystinuria; the rarity of the disease makes them, however, difficult to organize. 
